# Improved 18S rDNA profiling of parasite communities in salmonid tissues using a host blocking primer

**DOI:** 10.1007/s00436-024-08136-x

**Published:** 2024-02-06

**Authors:** Amanda L. Patchett, Megan L. Rigby, James W. Wynne

**Affiliations:** CSIRO Agriculture and Food, Hobart, Tasmania 7001 Australia

**Keywords:** 18S amplicon sequencing, Blocking primer, Salmonid, Parasite, Gill disease, *Neoparamoeba perurans*

## Abstract

Sensitive screening of eukaryotic communities in aquaculture for research and management is limited by the availability of technologies that can detect invading pathogens in an unbiased manner. Amplicon sequencing of *18S ribosomal DNA *(*rDNA*) provides a potential pan-diagnostic test to overcome these biases; however, this technique is limited by a swamping effect of host DNA on low abundance parasite DNA. In this study, we have adapted a host *18S rDNA* blocking assay to amplify eukaryotic DNA from salmonid tissue for amplicon sequencing. We demonstrate that effective salmonid *18S rDNA* blocking enables sensitive detection of parasite genera in salmonid gill swabs. Furthermore, 18S rDNA amplicon sequencing with host blocking identified enriched pathogen communities in gill swabs from Atlantic salmon suffering from severe clinical gill infections compared to those exhibiting no clinical signs of gill infection. Application of host *18S rDNA* blocking in salmonid samples led to improved detection of the amoebic parasite *Neoparamoeba perurans*, a parasite of significant threat to the Atlantic salmon aquaculture industry*.* These results reveal host *18S rDNA* blocking as an effective strategy to improve the profiling and detection of parasitic communities in aquaculture species. This assay can be readily adapted to any animal species for improved eukaryotic profiling across agricultural and veterinary industries.

**Supplementary Information:**

The online version contains supplementary material available at 10.1007/s00436-024-08136-x.

## Introduction

Parasitic infection remains a major cause of disease in agriculture, producing immense economic loss and impacting animal welfare. In the aquaculture sector alone, parasitism was estimated in 2015 to account for up to US $9.58 billion of annual global economic loss (Shinn et al. 2015). Teleost fish (such as salmonids) are susceptible to a diversity of parasitic infections at all life stages, including ciliates, amoeba, myxosporidians, flukes, tapeworms, roundworms and sea lice. Specifically, sea lice from the genera *Lepeophtheirus* and *Caligus*, and amoeba (*Neoparamoeba perurans*) are two examples of parasites that cause significant impacts to farmed salmonids globally (Costello 2009; Oldham et al. 2016). Parasites also co-exist in host tissues with other microorganisms, leading to disease caused by multiple or unknown aetiological agents. In salmonids, this includes complex gill disease (CGD) and nodular gill disease (NGD). However, despite the immense burden of parasitism on the aquaculture sector, few sensitive, feasible, and cost-effective methods exist for broadly examining parasite communities in animal tissues. Indeed, mainstay strategies for detection of parasitic infection typically involve techniques such as clinical observation, microscopy, serology or polymerase chain reaction (PCR). These are generally species-specific and limited by existing knowledge of invading pathogens (Momcilovic et al. 2019; Ricciardi and Ndao 2015). This lack methodology for community-based parasite detection in agriculture prevents broad infection screening, biases diagnoses and research towards well-investigated species, hinders novel species discovery, and does not allow for consideration of multiple aetiologies during complex disease diagnosis and research.

Surveillance of eukaryotic parasite communities in animal species can be achieved with the use of pan-parasite molecular tools, such as *18S ribosomal DNA* (*18S rDNA*) amplicon sequencing (Hino et al. 2016; Tanaka et al. 2014). Akin to *16S rDNA* amplicon sequencing of bacteria, this technique relies on cross-species detection of *18S rDNA* sequences in biological samples using universal primers. PCR-based gene amplification and sequencing is followed by computational analyses to assign sequences to their taxon of origin, or to identify novel pathogen sequences. While *18S rDNA* amplicon sequencing has the potential to rapidly profile parasite abundance and diversity, the sensitivity of this approach can be negatively impacted by the presence of eukaryotic host DNA with the sample. Indeed, samples such as biopsies or swabs often contain significant quantities of host DNA which, without depletion, will amplify with most universal 18S primers. Amplicon sequencing of such samples, unless performed at considerable read depth and therefore cost, tends to produce a swamping effect of host sequence reads, resulting in limited to no detection of low abundance parasitic *18S rDNA* (Liu et al. 2019; Belda et al. 2017).

The highly conserved nature of *18S rDNA* from vertebrate to microeukaryote species hinders the development of sufficient primers that universally bind to parasite *18S rDNA*, but not vertebrate host *18S rDNA*, for parasite-specific amplification. Instead, strategies to improve parasite identification by *18S rDNA* amplicon sequencing have focussed on the development of blocking primers that bind with high specificity to the host *18S rDNA* amplicon and low specificity to parasite amplicons (Leroux et al. 2022; Liu et al. 2019; Belda et al. 2017). These blocking primers interfere with host *18S rDNA* amplification by either preventing binding of universal primers to the DNA, or by blocking extension of the host template. Several blocking primer designs have been employed across various studies to block allele-specific DNA amplification. Commonly, primers are modified at 3’ ends by addition of short 3-carbon chains (C3 spacers), phosphate groups, inverted dT bases or amine groups, to prevent polymerase extension (Lee et al. 2011; Wang et al. 2013; Leroux et al. 2022; Liu et al. 2019). Other methods use peptide nucleic acid (PNA) clamps, which are synthetic DNA molecules with modified peptide backbones that anneal to target sequences and prevent amplification from upstream primers (Belda et al. 2017; Homma et al. 2022). The net effect is lowered PCR efficiency of host DNA amplification, allowing target DNA to be more readily detected by amplicon sequencing with low sequencing depth (Liu et al. 2019; Leroux et al. 2022; Belda et al. 2017; Homma et al. 2022).

Recent studies have successfully applied a host *18S rDNA* blocking strategy to profile eukaryotic communities in aquatic species including the whiteleg shrimp (*Litopenaeus vannamei*) and the flag cichlid (*Mesonauta festivus*) (Liu et al. 2019; Leroux et al. 2022). Here, we have optimised this strategy in salmonid species to detect and quantitate gill parasites using amoebic gill disease (AGD), caused by the marine protozoan parasite *Neoparamoeba perurans*, as a disease model. We demonstrate that application of a blocking primer with a C3 spacer to *18S rDNA* amplicon sequencing can partially suppress amplification of host DNA and successfully profile parasite diversity in mucosal gill swabs. Furthermore, detection of *N. perurans* using this method was correlated with species-specific real-time quantitative PCR. The assay described here facilitates assessment of parasite diversity in salmonid tissues, thereby providing a markedly less biased tool for investigation of complex or novel parasitic diseases. This assay can be adapted to host blocking across any animal species for improved parasite detection and complex disease diagnosis across the agricultural sector.

## Methods

### Biological samples

RNAlater preserved tissue (muscle) and swabs from laboratory reared Atlantic salmon (*Salmo salar*) were obtained from a previous study (Wynne et al. 2020). All procedures were approved by the Queensland CSIRO Animal Ethics Committee (project #2018–09) under the guidelines of the Australian Code for the Care and use of Animals for Scientific Purposes (2013). Atlantic salmon gill swabs (preserved in RNAlater) were obtained from commercially farmed Atlantic salmon from South-Eastern Tasmania, as approved by the Tasmanian Department of Primary Industries Water and Environment (DPIPWE) Animal Ethics Committee (AEC 04/2018–19). Ethanol preserved tissue samples from brown trout (*Salmo trutta*) and rainbow trout (*Oncorhynchus mykiss*) were also collected from hatchery reared animals in previous studies under approval from the DPIPWE Animal Ethics Committee (permits AEC 21/2011–12, AEC 20/2012–13). An in vitro cultured isolate of *Neoparamoeba perurans* was used in this study and is described in Botwright et al. (2020). Amoebae were cultured and harvested by centrifugation as described previously (Botwright et al. 2020). A schematic representation of the flow of experimental activities is included as Supplementary Fig. 1.

### DNA extraction

DNA was extracted from tissue samples and amoeba cell pellets using the DNeasy Blood and Tissue kit (QIAGEN, Germany) as per the manufacturer’s instructions. DNA was extracted from mucosal swab samples using the Wizard® Genomic DNA Purification Kit (Promega, USA) as previously described (Wynne et al. 2020). The quality and quantity of all extracted DNA was confirmed by a Nanodrop spectrophotometer.

### 18S primers

Universal *18S rDNA* amplification primers 1391F and EukBr with Illumina adaptor binding sequences, and *18S rDNA* blocking primer Salmonid_block_I-short_1391f, were designed based on priming regions reported in the Earth Microbiome Project 18S Illumina Amplicon Protocol (https://earthmicrobiome.org/protocols-and-standards/18s/) (Table 1) (Stoeck et al. 2010; Amaral-Zettler et al. 2009). Salmonid_block_l-short_1391f was adapted from Mammal_block_l-short_1391f with a single base-pair change from cytosine to tyrosine at base-pair 32, producing 100% homology with the salmonid *18S rDNA* sequence. This primer consists of two priming regions linked by a polydeoxyinosine linker to reduce melting temperature and prevent mispairing, and a C3 spacer at the 3’ end to prevent extension during PCR (Liu et al. 2019). The specificity of primer sequences for selected salmonid and parasitic *18S rDNA* was assessed by multiple sequence alignment using sequences obtained from the National Centre for Biotechnology Information (NCBI) and aligned using CLC Genomics Workbench version 21 (QIAGEN, Germany).

**Table 1:** Primer sequences for *18S rDNA* amplification with host blocking

Primer	Sequence (5’ – 3’)
1391F*	TCGTCGGCAGCGTCAGATGTGTATAAGAGACAGGTACACACCGCCCGTC
EukBr*	GTCTCGTGGGCTCGGAGATGTGTATAAGAGACAGTGATCCTTCTGCAGGTTCACCTAC
Salmonid_block_I-short_1391f	GCCCGTCGCTACTACCGATTGG/ideoxyI//ideoxyI//ideoxyI//ideoxyI//ideoxyI/TTAGTGAGGTCCT/3SpC3/

^*^Underlined sequence represents Illumina adaptor binding sites

### Quantitative polymerase chain reaction

Reduction of *18S rDNA* amplification by blocking primer in Atlantic salmon, brown trout and rainbow trout hosts was assessed using quantitative real-time PCR. Amplification of *18S rDNA* was performed from 20 to 30 ng of purified host DNA in 12.5 µl reactions using iQ SYBR Green Supermix (Bio-Rad, USA), according to the directions of the manufacturer. Controls with no DNA template were included. Forward and reverse primers were added at a final concentration of 0.2 µM each. Reactions were performed with or without blocking primer at various concentrations, as indicated in figure legends. Thermocycling was performed using a QuantStudio 5 thermocycler with an initial melting step at 94 °C for 3 min, followed by 35 cycles of 94 °C for 45 s, 65 °C for 15 s, 57 °C for 30 s and 72 °C for 90 s, and a final extension at 72 °C for 3 min. A melt curve was performed from 60 to 95 °C to confirm specific product amplification.

A plasmid standard of Atlantic salmon *18S rDNA* was created from this amplicon using the pGEM-T-easy (Promega, USA) vector system as per the manufacturer’s instructions. Plasmid was purified using Pure Yield plasmid purification kit (Promega, USA) and sequenced using M13 primers to confirm identity. The plasmid was used to generate a standard curve by tenfold dilution to assess amplification efficiency and impute DNA copy number of unknown samples. Dose–response relationships were assessed by one- or two-way ANOVA in R version 4.2.1 using the base statistics package. Plots were generated using ggplot2 version 3.3.6.

### End-point polymerase chain reaction

Amplification of *18S rDNA* was performed from 20 to 30 ng of appropriate template DNA in 20 µl reactions using Platinum Taq DNA Polymerase (Bio-Rad, USA), according to the directions of the manufacturer. Controls with no DNA template were included. Forward and reverse primers were added at a final concentration of 0.25 µM each. Reactions were performed with or without blocking primer at 1.6 µM, as indicated in figure legends. Thermocycling was performed with an initial melting step at 94 °C for 3 min, followed by 25 cycles of 94 °C for 45 s, 65 °C for 15 s, 57 °C for 30 s and 72 °C for 90 s, and a final extension at 72 °C for 3 min. PCR amplicons were visualised by gel electrophoresis to confirm *18S rDNA* amplification.

### Amplicon sequencing

*18S rDNA* amplicons prepared by end-point PCR were sequenced by 300 base-pair, paired-end sequencing on the Illumina MiSeq platform, by the Australian Genome Research Facility (AGRF, Australia). Raw sequencing data is available in the NCBI Sequence Read Archive (SRA) at BioProject PRJNA947667, accessions SRR23942186-SRR23942208. Paired-end sequencing reads were merged using FLASH version 2.2.0, based on a minimum overlap of 60 base-pairs (Magoc and Salzberg 2011). For each merged read, the primer sequences were trimmed and low-quality reads were removed based on a quality score of 20 using USEARCH version 11.0.667 (Edgar 2010).The fastq file for each sample was then converted to a fasta, and as a further quantity control, reads with a sequence length greater than 160 base-pair were removed using mothur version 1.43.5 (Schloss et al. 2009). The individual (merged) sequence reads for each sample were then combined into a single fasta file and the entire dataset was dereplicated using VSEARCH version 2.21.1 (Rognes et al. 2016). The dereplication step removes redundant (identical) sequence reads to generate a single fasta file containing only unique sequence reads. These unique sequences were denoised using USEARCH version 11.0.667 (Edgar 2010) to generate a final set of ZOTUs (zero-radius operational taxonomic units). Next, the final set of ZOTUs were taxonomically classified using the SILVA 18S database NR v138 within QIIME version 2021.4 (Caporaso et al. 2010). Sequences were only classified if the confidence level was > 0.5 using VSEARCH. A ZOTU count table was then created quantifying the raw number of sequences reads that are 99% identical to the ZOTU sequence. This was achieved using the usearch_global command within USEARCH tool (Edgar 2010). Finally the detected taxa were rarefied to 65,000 reads, which was below the lowest read count observed. The most abundant unique sequences were plotted at the genus level using phyloseq version 1.40.0 in R version 4.2.1 (McMurdie and Holmes 2013). Other plots were generated using ggplot2 version 3.3.6 (Wickham 2009) and statistics calculated by paired Student’s *t-*test using the R version 4.2.1 base statistics package.

## Results

### Identification of a blocking primer specific to salmonid 18S rDNA

**Fig. 1:**
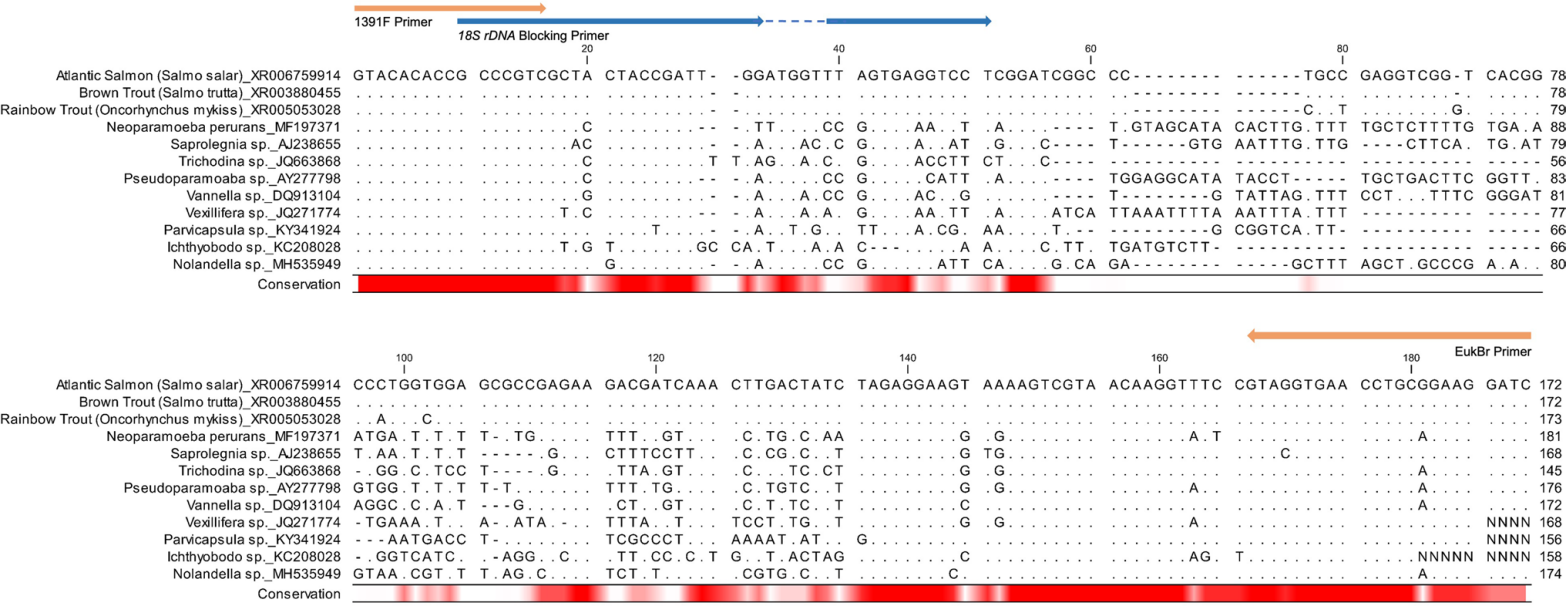
Alignment of *18S rDNA* amplicons across salmonid and gill-associated microeukaryote species**. ***18S rDNA* sequences for salmonid and associated parasitic species were obtained from the NCBI GenBank database and trimmed at primer binding sites (orange arrows) to produce amplicon sequences. Sequences were aligned against the Atlantic Salmon *18S rDNA* amplicon. Conserved bases are represented by (.), sequence gaps by (-) and ambiguous bases by (N). The blue arrow demonstrates the binding of the salmonid *18S rDNA* amplification blocking primer. Sequence conservation is indicated from high to low on a gradient of red to white

Sensitive detection of low abundance parasite *18S rDNA* in host tissue is hindered by “swamping” effects from host *18S rDNA*. To increase the sensitivity of *18S rDNA* amplicon sequencing to detection of parasite DNA in salmonid tissue, we used an *18S rDNA* blocking primer containing five internal deoxyinosine bases and 3’ C3 spacer modification. This primer was modified by a single base compared to the recommended blocking primer sequence reported in the Earth Microbiome Project 18S Illumina Amplicon Protocol (https://earthmicrobiome.org/protocols-and-standards/18s/), to be 100% identical to Atlantic salmon (*S. salar*), brown trout (*S. trutta*), and rainbow trout (*O. mykiss*) DNA*.* Alignment of predicted *18S rDNA* amplicon sequences demonstrates complete DNA conservation at the blocking primer binding site across the three salmonid species (Fig. 1). Comparatively, a 23% to 40% difference in *18S rDNA* sequence is observed at the blocking primer site in relevant salmonid parasite species. These nucleotide differences occurred mostly at the 3’ end of the blocking primer and are predicted to reduce the affinity of blocking primer binding to parasite *18S rDNA* compared to salmonid hosts.

### 18S rDNA blocking specifically reduces salmonid DNA amplification

**Fig. 2:**
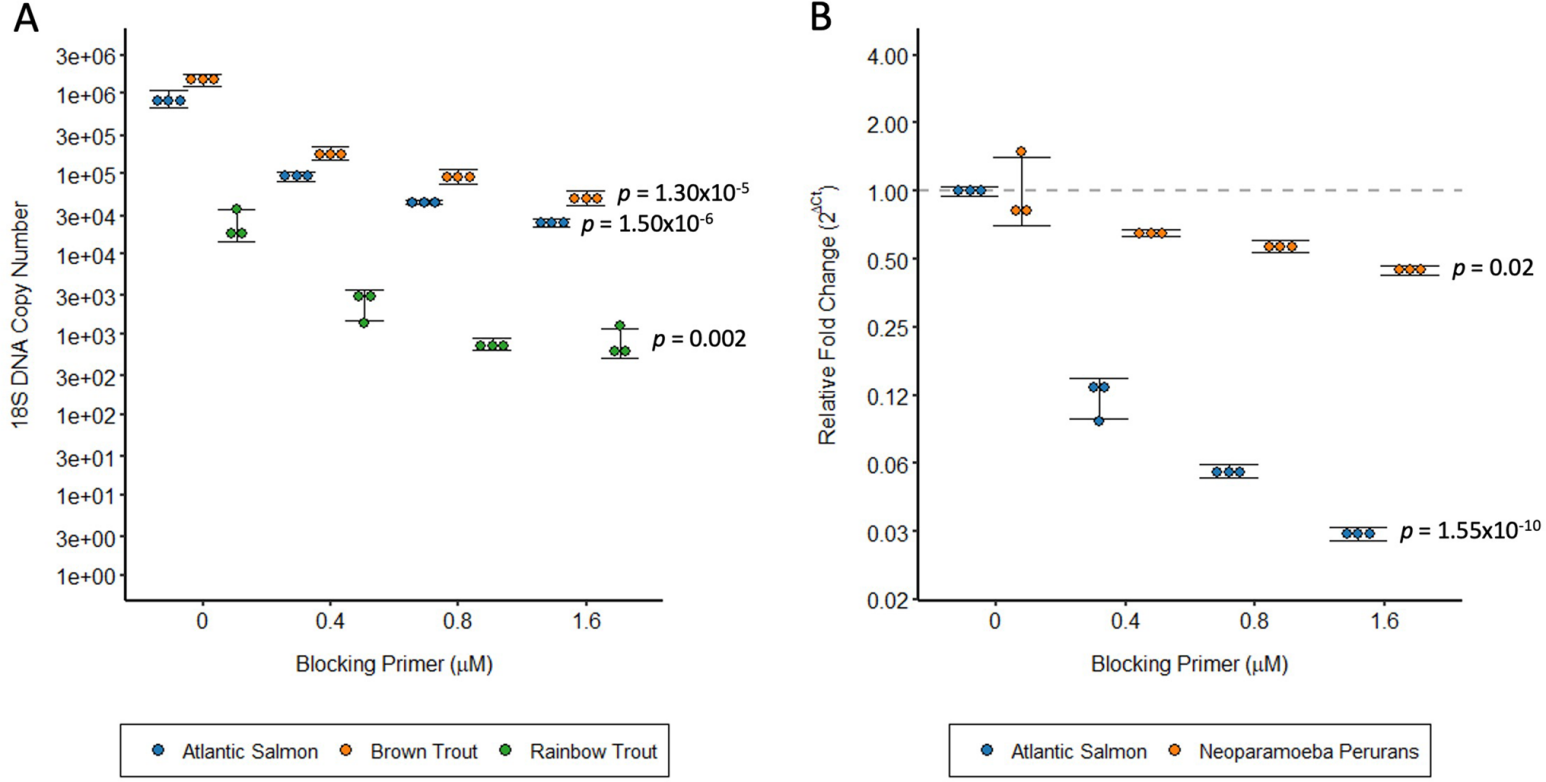
Reduced salmonid *18S rDNA* PCR amplification with use of a blocking primer. *18S rDNA* was amplified by qPCR from DNA samples obtained from Atlantic salmon (*Salmo salar*), brown trout (*Salmo trutta*), rainbow trout *(Oncorhynchus mykiss*) or *Neoparamoeba perurans, *with and without salmonid *18S rDNA* blocking primer at 0.4, 0.8 and 1.6 µM. (A) Absolute amplicon copy number was calculated from a linear curve of known DNA standards. (B) *18S rDNA* amplification was calculated by change in Ct relative to an unblocked control. Error bars represent standard deviation of triplicate reactions (points). Statistical significance of dose–response effects was calculated by one-way ANOVA

To determine whether the blocking primer can be used to specifically reduce amplification of host salmonid *18S rDNA*, we performed qPCR across a concentration gradient of blocking primer (0–1.6 µM). Template DNA was obtained from Atlantic salmon, brown trout, rainbow trout and *N. perurans* as model salmonid and parasite species. Comparison of *18S rDNA* blocking across salmonid species revealed significant concentration-dependant decreases in *18S rDNA* amplification of around 29-fold in brown trout and rainbow trout (*p* = 1.50 × 10^–6^ and *p* = 0.002, respectively), and 35-fold in Atlantic salmon (*p* = 1.30 × 10^–5^; Fig. 2a) at the 1.6 µM concentration. A secondary experiment comparing Atlantic salmon *18S rDNA* blocking to the parasite *N. perurans* revealed a similar 33-fold decrease in salmonid *18S rDNA* amplification relative to an unblocked control (*p* = 1.55 × 10^–10^; Fig. 2b) at the 1.6 µM concentration*.* In contrast, amplification of *N. perurans 18S rDNA* decreased only twofold with blocking primer (*p* = 0.02). A statistically significant difference in the effect of blocking primer on *18S rRNA* amplification between salmon and *N. perurans* was measured by two-way ANOVA (*p* = 4.29 × 10^–14^). Together these results suggest that *18S rDNA* blocking primer specifically lowers salmonid amplification efficiency of *18S rDNA* compared to parasite DNA.

### 18S blocking primer increases sensitivity of N. perurans detection in gill swabs

**Fig. 3:**
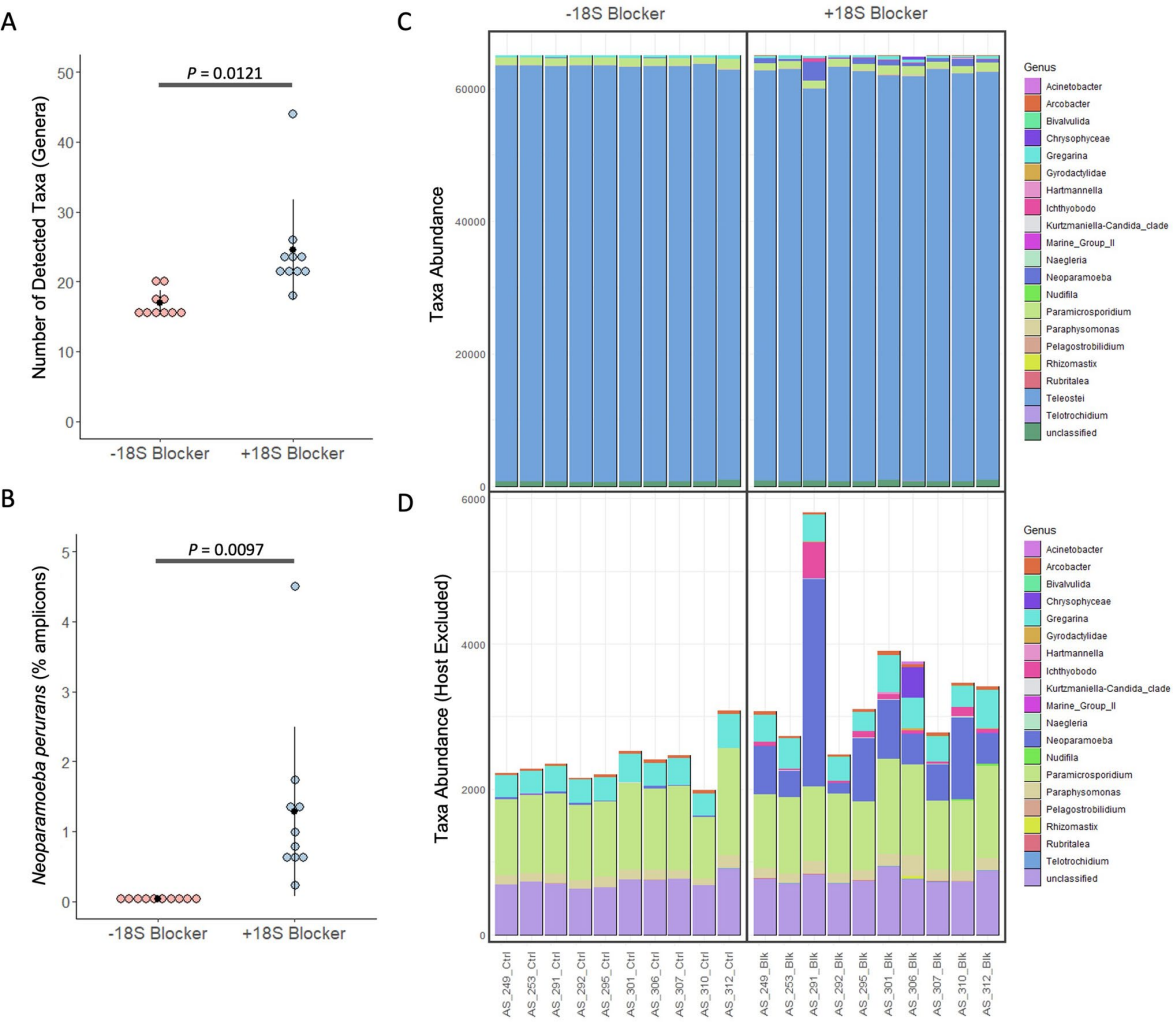
Increased sensitivity of parasite detection by *18S rDNA* amplicon sequencing with use of a blocking primer. *18S rDNA* was sequenced in gill DNA swabs obtained from experimentally *Neoparamoeba perurans-*infected Atlantic salmon (*Salmo salar*)*, *amplified with and without salmonid *18S rDNA* blocking primer (1.6 µM). Detected *18S rDNA* amplicons were assigned taxa based on DNA sequence. (A) The number of detected taxa per sample was summarised at the genus level and compared between samples amplified with and without blocking primer. (B) The percentage of total amplicons assigned to *N.* *perurans* was calculated and compared between samples amplified with and without blocking primer. *P-*values were calculated using a paired Student’s *t*-test. (C-D) Detected taxa was rarefied to 65,000 amplicons and visualised across samples with (C) salmonid *18S rDNA* amplicons included and (D) salmonid *18S rDNA* amplicons excluded

Salmonid *18S rDNA* blocking primer specifically reduced host DNA amplification, revealing *18S rDNA* amplicon sequencing as a potential strategy for profiling of parasite communities in salmonid tissues. To determine whether salmonid *18S rDNA* blocking can enhance parasite detection in Atlantic salmon gill swabs, we applied our assay to swabs from ten animals experimentally infected with *N. perurans* in a pervious study (Wynne et al. 2020)*.* Amplification and sequencing of *18S rDNA* was performed on each sample with and without blocking primer. Rarefraction analysis at the OTU level demonstrated that the addition of the blocking primer increased the OTU diversity within gill swabs (Supplementary Fig. 2A). Subsequent rarefication of the read depth to 65,000 reads did not significantly affect OTU diversity (Supplementary Fig. 2B). Analysis of the number of genera detected in each sample by amplicon sequencing revealed an average 6.7 count increase when salmonid blocking primer was included in *18S rDNA* amplification (*p* = 0.012; Fig. 3a). Furthermore, analysis of the percentage of amplicons assigned to our model parasite taxon *N. perurans* revealed a 1.2% increase in detected amplicons relative to matched unblocked controls (*p* = 0.0097; Fig. 3b). Visualization of taxa abundance further demonstrated the increase in parasite amplicon detection with salmonid *18S rDNA* blocking, although this remained somewhat masked by high levels of host amplification (Fig. 3c). Removal of salmonid-specific reads from the plot allowed the abundance of other microeukaryotes to be more easily visualised, with notable improvements in detection of low abundance genera such as *Neoparamoeba*, *Ichthyobodo*, and *Chrysophyceae* with salmonid *18S rDNA* blocking (Fig. 3d). Other genera, such as *Gregarina* and *Paramicrosporidium,* demonstrated consistent abundance with salmonid *18S rDNA* blocking.

### 18S amplicon sequencing with blocking primer measures burden of N. perurans infection in gill swabs

**Fig. 4:**
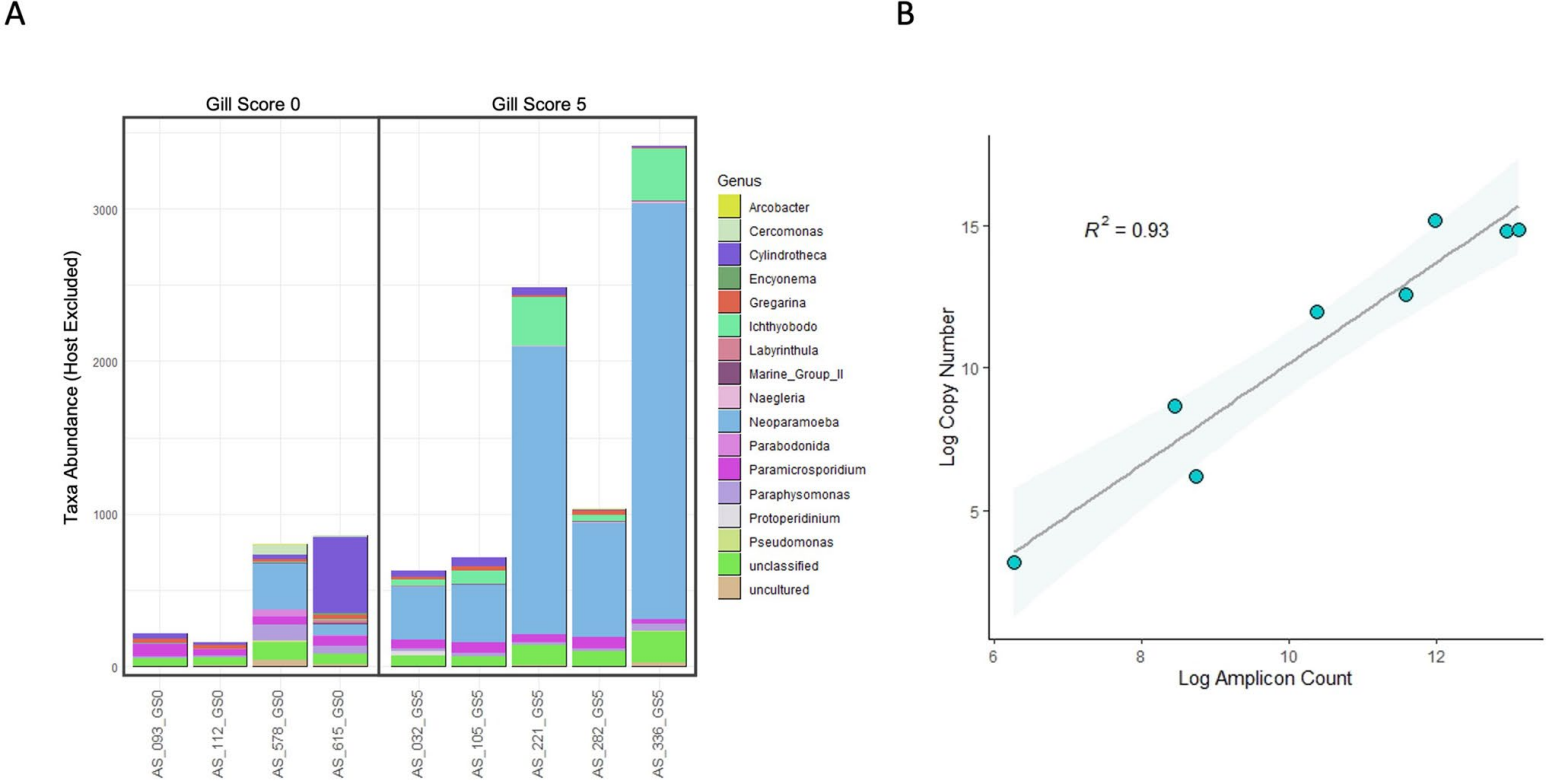
*18S rDNA* amplicon sequencing with blocking primer accurately quantifies *N.* *perurans* infection. *18S rDNA* was sequenced in gill DNA swabs obtained from farmed Atlantic salmon (*Salmo salar*) and selected based on clinical gill score (0 to 5; 0 indicates no clinical signs of infection, 5 indicates most severe clinical infection). All amplifications were performed with salmonid *18S rDNA* blocking primer (1.6 µM). Detected *18S rDNA* amplicons were assigned taxa based on DNA sequence. (A) Detected taxa was rarefied to 5000 amplicons and visualised across samples with salmonid *18S rDNA* amplicons excluded. (B) *N.* *perurans 18S rDNA* was quantified in the same samples by qPCR and absolute copy number was calculated based on known DNA standards. *18S rDNA* copy number was compared with amplicon count, excluding a single sample with no *N.* *perurans* detected. Correlation was assessed by linear regression and coefficient of determination (R^2^), with confidence intervals indicated by the shaded area

Detection of *N. perurans* infection from salmonid gill swabs by *18S rDNA* amplicon sequencing was enhanced with the use of a host DNA blocking primer. To determine whether this assay can be used to measure the level of *N. perurans* infection, we amplified and sequenced *18S rDNA* with blocking primer in gill swabs from nine commercially farmed Atlantic salmon. These salmon demonstrated either no clinical signs of infection (gill score 0) or most severe clinical infection (gill score 5). Visualisation of taxa abundance after removal of salmonid-specific reads revealed greater abundance of *N. perurans* in swabs obtained from high scoring gills, consistent with severe infection (Fig. 4a). However, this abundance varied drastically across the samples. Furthermore, *N. perurans* was detected in two samples with no clinical signs of infection. To determine whether *N. perurans 18S rDNA* abundance measured by amplicon sequencing is truly reflective of parasite DNA levels in gill swabs, we detected *N. perurans 18S rDNA* in the same samples using absolute qPCR, and correlated PCR copy number with amplicon count from the 18S sequencing analysis. All samples within this analysis received the blocking primer. These findings revealed a linear correlation between *N. perurans* infection quantitated by qPCR and amplicon sequencing (*R*^2^ = 0.93; Fig. 4b). Together these findings suggest that amplicon sequencing with host *18S rDNA* blocking primer can effectively measure parasite burden in salmonid samples, and may detect parasite colonisation at the gill prior to symptom onset.

## Discussion

Gill diseases, such as complex gill disease (CGD) and nodular gill disease (NGD), have emerged as significant health burdens affecting farmed salmonids globally. These conditions are typically characterised by multifactorial disease aetiologies that may include viral, bacterial and parasitic pathogens (Herrero et al. 2018; Boerlage et al. 2020). *N. perurans* has previously been identified in the presence of a plethora of additional pathogenic parasites and bacteria and is a known contributor to CGD cases (Boerlage et al. 2020; Herrero et al. 2018). These coinfections include the harmful microsporidian parasite *Desmozoon lepeophtherii*, which has been identified alongside *N. perurans* within marine life stages, producing a mortality rate of up to 80% (Herrero et al. 2018). Similarly, multiple amoeba species have been associated with NGD in rainbow trout including *Vannella* sp., *Naegleria* sp., *Protacanthamoeba* sp., *Acanthamoeba* sp. and *Hartmannella* sp., however the true aetiological agents remain to be resolved (Dykova et al. 2010; Padros and Constenla 2021; Perolo et al. 2019; Quaglio et al. 2016; Speare 1999). Despite the impact of gill disease on farmed salmonids, few studies consider the impacts of species co-colonisation when understanding disease progression and developing interventions. Considering the often complex and multifactorial nature of infection, it is important that parasite detection methodologies capture the diversity of eukaryotic insults.

Mainstay techniques for parasite detection in animal species are limited in their ability to capture holistic and unbiased knowledge of infiltrating eukaryotic communities. This increases the likelihood of misdiagnoses, does not allow entire eukaryote communities to be considered in treatment and research decisions, and can prevent novel and complex cases from being identified. For example, classical detection by microscopy does not resolve pathogens to the species-specific level, is not scalable for large-scale pathogen screening, and relies on knowledge of the pathogenic parasite by the diagnostician, leading to diagnostic biases (Ricciardi and Ndao 2015; Momcilovic et al. 2019). Taxonomic classification based on gross morphology alone is particularly difficult for many amoeba species due to their inherent plastic morphology (Dykova and Lom 2004). Host serology and PCR provide more definitive techniques for detection; however, these technologies require existing knowledge of specific antigens to be informative at the species-specific level. Furthermore, their targeted nature prevents broad screening, potentially leading to false negative results or misdiagnosis of causative agents. Serology is also limited by its ability to differentiate current from previous parasite infiltration due to the long-lived nature of specific immunoglobulins after infection (Ricciardi and Ndao 2015; Momcilovic et al. 2019).

The recent development of *18S rDNA* blocking primers that specifically decrease the efficiency of host DNA PCR amplification has increased capacity in parasite detection by allowing broad and sensitive cross-species screening via *18S rDNA* profiling (Leroux et al. 2022; Liu et al. 2019). By reducing host *18S rDNA* amplification, these blocking primers successfully prevent “swamping” of low abundance parasite reads by host reads in mixed samples where high *18S rDNA* homology prevents development of species-specific primers. Its important to note that the C3 spacer blocking primer used in our study does not completely inhibit host amplification. Although our qPCR results demonstrate a 33-fold decrease in host amplification when using the blocking primer, most of the sequence reads remained derived from the host. Other studies have reported a range of blocking efficiencys when using C3 spacer blocking primers. For instance, Liu et al (2019) found that blocking primers with C3 spacers on the 3’ end reduced host amplification of shrimp DNA by 99%, but oyster DNA by only 17%. Similarly, a C3 spacer against canine mitochondrial DNA reduced host amplification by only 25% (Huggins et al. 2020). More recent studies have shown that PNA clamp blocking primers may be more effective than C3 spacer blockers. Indeed, Homma et al (2022) showed that a PNA clamp suppressed 99.3–99.9% of fish DNA amplification, whereas the blocking primer suppressed 3.3–32.9%. Even though our C3 blocking primer did not completely remove host amplification, we demonstrate here that parasitic DNA is enriched when the blocking primer is used. It is also possible that blocking efficiency will be affected by choice of host tissue samples. For instance, we hypothesise that blocking efficiency will be highest for samples that contain the least concentration of host DNA (i.e., gut samples). While our study did not examine this aspect, we recognize that future research will be required if microeukaryote abundance is to be compared across different host tissues.

We have demonstrated substantial host blocking of salmonid *18S rDNA* in our study, allowing community profiling of microeukaryotes to be more effectively performed in Atlantic salmon gill swabs. Moreover, amplicon sequencing with a blocking primer effectively quantified the abundance of pathogenic *N. perurans,* demonstrating potential use for understanding relative abundance of single species across tissue samples. Notably, the detection of some parasite genera including *Paramicrosporidium* and *Gregarina* was unchanged with the use of blocking primer. This suggests that differences in parasite *18S rDNA* sequences and baseline burden may affect the affinity of primer binding, and alter which species exhibit greater amplification efficiency in this assay. This potential difference in the efficiency of parasite amplification indicates that care should be taken when using amplicon counts for quantitating true parasite abundance. As a qualitative detection tool however, our findings indicate that with careful blocking primer design, amplicon sequencing with host *18S rDNA* blocking primer provides an effective assay for parasite identification.

The *18S rDNA* blocking assay described here provides a solution for more rapid and feasible analysis of the contributions of various pathogenic species to diseases such as CGD and NGD in salmonid aquaculture. In AGD cases, although *N. perurans* is regarded as the primary causative agent (Crosbie et al. 2012), it is known that a range of other amoebae co-colonise the AGD-affected gill (English et al. 2019). A pathogenic role for these other amoebae is unclear, however in vivo Atlantic salmon challenges with cultured parasite have suggested that they represent either normal eukaryotic gill fauna or opportunistic colonisation (English et al. 2021). Further studies are required to confirm this finding in the context of natural disease progression. Current strategies for detection of* N. perurans* in farmed salmonids generally rely on either gross morphological detection of typical AGD patches of white and swollen tissue, histology of wet gill mounts, or non-invasive qPCR-based detection (Adams et al. 2004; Downes et al. 2017). These techniques fail to understand the full diversity of parasites present during AGD infection. In comparison, our *18S rDNA* amplicon sequencing has successfully identified a range of amoebic and non-amoebic parasitic genera present on AGD-affected gills. This blocking assay will allow more rapid and sensitive detection of *N. perurans* in the context of the gill community in future studies, allowing parasitic relationships to be elucidated and accelerating research in this field beyond single parasite detection for improved NGD and CGD management.

Our findings demonstrate successful profiling of eukaryotic communities in gill swabs with a sequencing read coverage that was approximately 6000 reads per sample at its lowest, and around 25,000 reads per sample on average. This allowed many samples to be profiled in the same sequencing run, demonstrating the cost-effective nature of the assay for use across a variety of applications and sectors, including the aquaculture sector. Notably, the sensitivity of our assay was sufficient to detect low levels of colonisation with our model parasite *N. perurans* in Atlantic salmon gill swabs before clinical signs of AGD were evident (gill score = 0). Further sampling and studies are required to determine whether this pre-clinical detection is of use for improved parasitic management in the farmed salmonid environment. Nonetheless, this early parasite detection using *18S rDNA* blocking could enable the course of gill disease to be more easily understood for rational design of parasite interventions in both salmonids and other species.

In this study, we have applied *18S rDNA* amplicon sequencing to detect known parasitic genera in a well characterised gill disease. However, the approach described here can also be used to explore the aetiology of novel and emerging gill diseases, where multiple parasites may play a role. One such example is NGD in freshwater farmed rainbow trout from Europe, where the causative agent(s) remain unclear. Indeed, a diversity of amoeba species have been identified in NGD affected gills including *Acanthamoeba*, *Hartmannella*, *Naegleria*, *Protacanthamoeba* and *Vannella* (Dykova et al. 2010). Our future research will apply this assay to NGD samples to help resolve this disease aetiology. Indeed, the universal nature of *18S rDNA* primers enables this assay to be applied across a myriad of animal species and diseases, allowing for holistic, integrated and universal parasite detection, discovery and analysis across many research and agricultural spaces.

## Data availability

Amplicon sequencing data is available in the NCBI Sequence Read Archive (SRA) in FASTQ format at BioProject PRJNA947667, accessions SRR23942186-SRR23942208. Other datasets generated during the current study are available from the corresponding author on reasonable request.

### Supplementary Information

Below is the link to the electronic supplementary material.

Supplementary file1 (PDF 192 KB)

## Declarations

Ethical approval.

All procedures were approved by the Queensland CSIRO Animal Ethics Committee (project #2018–09) or the Tasmanian Department of Primary Industries Water and Environment (DPIPWE) Animal Ethics Committee (permits AEC 21/2011–12, AEC 20/2012–13,AEC 04/2018–19) under the guidelines of the Australian Code for the Care and use of Animals for Scientific Purposes (2013).
