# Supplementary material for: Improved 18S rDNA profiling of parasite communities in salmonid tissues using a host blocking primer
Source: Parasitol Res. 2024 Feb 6;123(2):124. doi: 10.1007/s00436-024-08136-x (PMC10847071; doi:10.1007/s00436-024-08136-x)
Supplement: Supplementary file 1 — Supplementary file1 (PDF 192 KB) [file 436_2024_8136_MOESM1_ESM.pdf]

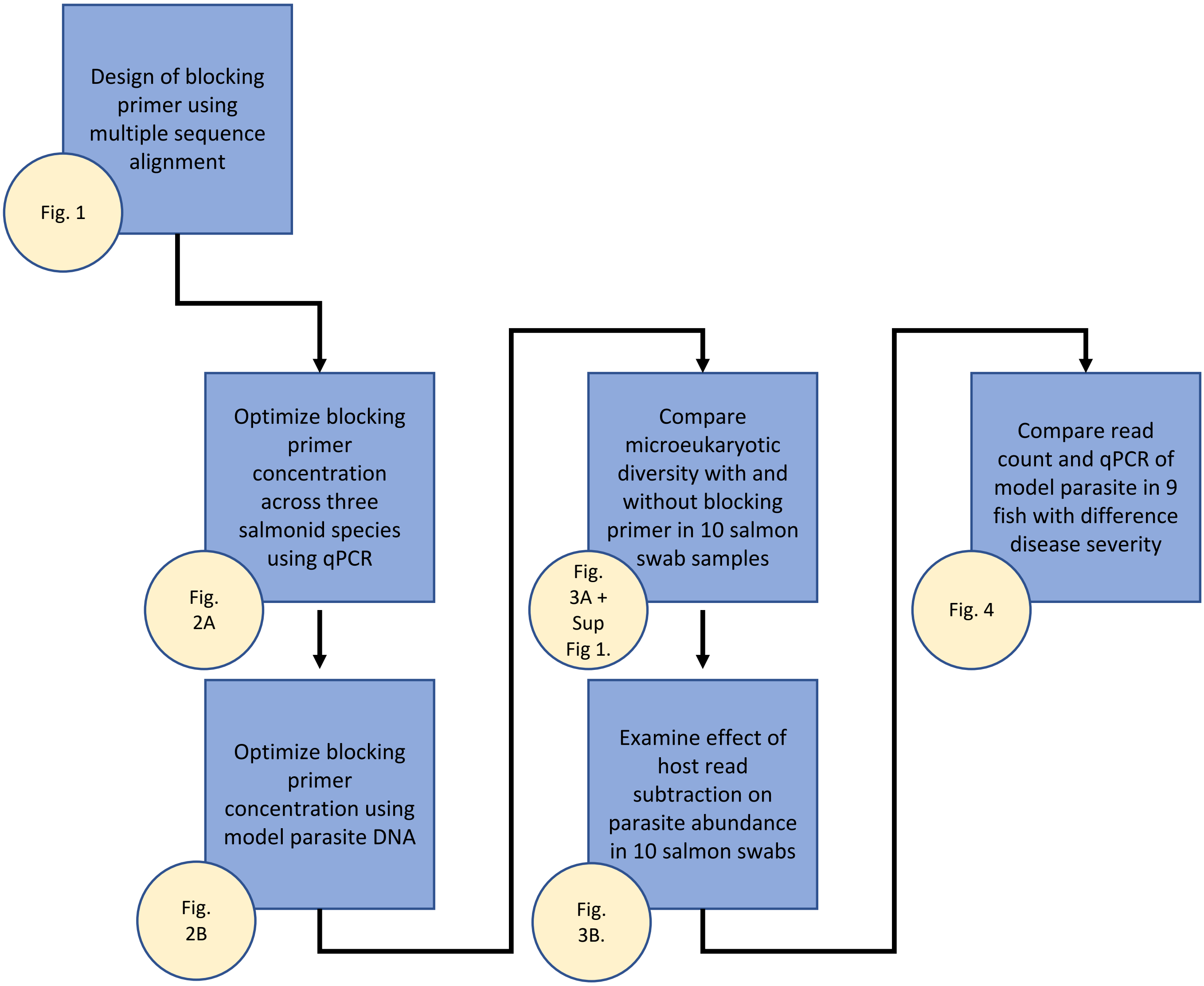

Supplementary Figure 1. Experimental workflow from the design and optimization of the blocking primer to the application in diseased gill swabs for quantifying parasite load compared to qPCR. The representative figures for each experimental activity is illustrated in the yellow circles.

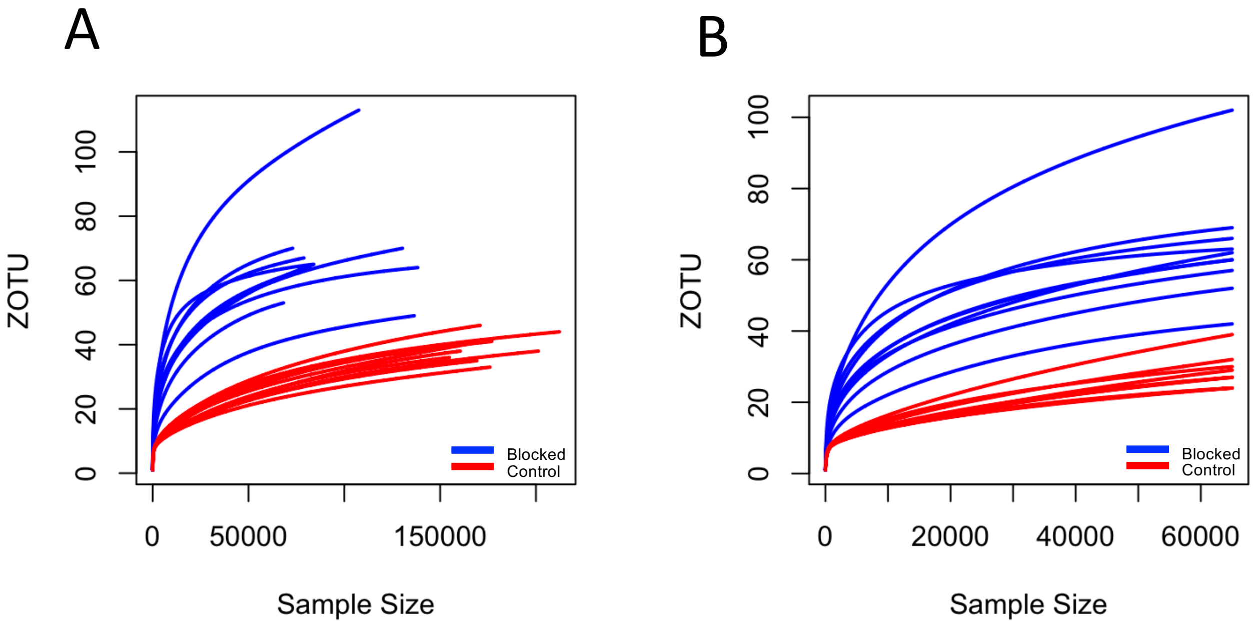

Supplementary Figure 2. A) Rarefraction curves for 18S rDNA OTUs with increasing read depth (Sample size) for 10 Atlantic salmon swab samples with (*blue lines*) and without (*red lines*) blocking primer. B) rarefaction curve for the same 10 swabs samples following rarefication to 65,000 reads.
